# Spatial Variation in the Population Structure and Reproductive Biology of *Rimicaris hybisae* (Caridea: Alvinocarididae) at Hydrothermal Vents on the Mid-Cayman Spreading Centre

**DOI:** 10.1371/journal.pone.0060319

**Published:** 2013-03-29

**Authors:** Verity Nye, Jonathan T. Copley, Paul A. Tyler

**Affiliations:** Ocean and Earth Science, National Oceanography Centre, University of Southampton, Southampton, United Kingdom; Aristotle University of Thessaloniki, Greece

## Abstract

The dynamics and microdistribution of faunal assemblages at hydrothermal vents often reflect the fine-scale spatial and temporal heterogeneity of the vent environment. This study examined the reproductive development and population structure of the caridean shrimp *Rimicaris hybisae* at the Beebe and Von Damm Vent Fields (Mid-Cayman Spreading Centre, Caribbean) using spatially discrete samples collected in January 2012. *Rimicaris hybisae* is gonochoric and exhibits iteroparous reproduction. Oocyte size-frequency distributions (21-823 µm feret diameters) varied significantly among samples. Embryo development was asynchronous among females, which may result in asynchronous larval release for the populations. Specimens of *R. hybisae* from the Von Damm Vent Field (2294 m) were significantly larger than specimens from the Beebe Vent Field. Brooding females at Von Damm exhibited greater size-specific fecundity, possibly as a consequence of a non-linear relationship between fecundity and body size that was consistent across both vent fields. Samples collected from several locations at the Beebe Vent Field (4944–4972 m) revealed spatial variability in the sex ratios, population structure, size, and development of oocytes and embryos of this mobile species. Samples from the Von Damm Vent Field and sample J2-613-24 from Beebe Woods exhibited the highest frequencies of ovigerous females and significantly female-biased sex ratios. Environmental variables within shrimp aggregations may influence the distribution of ovigerous females, resulting in a spatially heterogeneous pattern of reproductive development in *R. hybisae*, as found in other vent taxa.

## Introduction

Deep-sea chemosynthetic environments supporting chemosynthesis-based faunal assemblages are distributed widely but patchily throughout the global ocean.

Reproduction is therefore an essential process for the establishment and maintenance of isolated populations of specialist vent, seep and whale-fall fauna. Understanding the life histories of organisms inhabiting these insular environments is a prerequisite for understanding their ecology, population biology, dispersal, gene flow and biogeography [1], [2], [3], [4].

More than 400 new faunal species have been described from deep-sea hydrothermal vents since the 1970s [5], and aspects of life-history biology have been elucidated in more than 90 species from vents, seeps, and whale falls (Copley, Nye et al., unpublished data). Studies have described a variety of reproductive traits and developmental modes in species from chemosynthetic environments, and revealed spatial and temporal patterns in the reproductive development of some species (see [2], [6] for reviews).

The Mid-Cayman Spreading Centre (MCSC) is an ultraslow-spreading and geographically isolated ridge in the Caribbean that hosts two high-temperature hydrothermal vent fields [7]. The Beebe Vent Field (∼4960 m) is situated on the axis of the MCSC and it consists of a sulfide mound (∼80 m diameter, 50 m height) surmounted by several active black-smoker chimney complexes and areas of diffuse flow [7]. The Von Damm Vent Field (∼2300 m) is a conical mound (∼150 m diameter, 70 m height) venting clear, buoyant fluids, located off-axis (approximately 13 km away from the Beebe Vent Field) on the upper slopes of the Mount Dent oceanic core complex [7].

Research efforts on the fauna at MCSC vents have so far focused on the taxonomy, phylogenetics and assemblage compositions. The Beebe vent assemblage includes provannid gastropods, anemones and ophiuroids, whereas the faunal assemblage at the Von Damm Vent Field includes skeneid gastropods, hippolytid shrimp, lysianssid amphipods and tubeworms [7], [8], [9], [10]. The alvinocaridid shrimp *Rimicaris hybisae* [8] is present and abundant at both known MCSC vent fields [8].

To date, more than 125 species representing 33 families of decapods have been reported from deep-sea chemosynthetic environments [11], yet the reproductive traits of only ten species have been described [12], [13], [14], [15], [16], [17], [18]. Reproductive patterns of decapods from chemosynthetic environments are thought to have strong phylogenetic constraints [2], [12].

The family Alvinocarididae [19] is represented to date by 26 described species from eight genera and appears to be endemic to deep-sea chemosynthetic environments [8]. Alvinocaridid shrimp examined previously exhibit planktotophic development and gametogenesis characteristic of carideans [12], [13], [15], [16], [17]. Seasonal reproduction has been described in *Alvinocaris stactophila* [20] from the Brine Pool cold seep (650 m) in the Gulf of Mexico, where the seasonal peak in surface productivity and its export may be a cue for larvae to hatch [15].

Zonation in the population structure and reproductive biology of *Alvinocaris stactophila* has also been revealed at the Brine Pool ([15], Nye, unpublished data). Avoidance of sulfidic extremes by female crustaceans brooding embryos has been proposed for several taxa at vents and seeps (e.g., [14], [15], [18], [21], [22]). A similar explanation has been invoked to explain the apparent scarcity of ovigerous females of *Rimicaris exoculata* [23] in the immediate vicinity of black smokers at deep vents on the Mid-Atlantic Ridge (MAR) [13], [24].

The aims of this study were therefore to: (1) examine variation in the population structure and reproductive features of *Rimicaris hybisae* between the Beebe and Von Damm vent fields; (2) assess spatial variation in the reproductive features of *R. hybisae* within the Beebe Vent Field; (3) discuss and compare the results with data available for other alvinocaridid species. This is the first study on the autecology of vent fauna from the Mid-Cayman Spreading Centre, and reveals a high degree of spatial variability in the population structure and reproductive features of this mobile species in the vent environment.

## Materials and Methods

To assess spatial variation in population structure and reproductive features, samples of *Rimicaris hybisae* were collected from two vent fields at the Mid-Cayman Spreading Centre, Caribbean, during the 18^th^ voyage (16^th^ leg) of the RV ‘Atlantis’ (see Table 1). Samples were collected using a suction sampler attached to the remotely operated vehicle (ROV) ‘Jason II’. Four samples were collected from different locations within the Beebe Vent Field (4944–4972 m; Table 1): J2-613-24 was collected from a large, high-density aggregation of shrimp at the base of a chimney; J2-619-15 was collected from a large, high-density aggregation of shrimp at the edge of a gulley; J2-613-19 was taken from a small, dense aggregation of shrimp, next to anemones, provannid gastropods and bacterial mats; J2-620-32 was taken in a peripheral area, dominated by anemones with sparse shrimp.

**Table 1:** *Rimicaris hybisae*: Sample and population data for 959 specimens used in this study.

Sample no.	Cruise	Sample method	Vent Field	Location	Depth (m)	Latitude (N)	Longitude (W)	Date (JD)	Total no. specimens	Males			Females				Sex Ratio TM:TF	χ^2^ (1 df)	Significance
										Total	M	SM	Total	F	BF	HF			
J2-613-24	Atlantis 18_16	Jason II	Beebe	Beebe Woods	4971	18.546182	81.718086	12/01/2012	254	84	32	52	170	37	108	25	0.49∶1	28.44	***
J2-619-15	Atlantis 18_16	Jason II	Beebe	Shrimp Gulley	4944	18.546563	81.717705	22/01/2012	118	48	36	12	70	42	28	0	0.69∶1	3.74	NS
J2-613-19	Atlantis 18_16	Jason II	Beebe	Beebe Woods	4972	18.546974	81.718339	11/01/2012	96	44	22	22	52	47	5	0	0.85∶1	0.51	NS
J2-620-32	Atlantis 18_16	Jason II	Beebe	Beebe Woods	4964	18.546929	81.718278	23/01/2012	94	76	48	28	18	16	0	2	4.2∶1	34.56	***
			Beebe	All					562	252	138	114	310	142	141	27	0.81∶1	5.78	*
J2-617-5/8	Atlantis 18_16	Jason II	Von Damm	Spire	2294	18.376630	81.798143	19/01/2012	397	141	103	38	256	147	77	32	0.55∶1	32.74	***
								Total	959	393	241	152	566	289	218	59	0.69∶1	30.85	***

BF, brooding female; F; female without brooding or recently hatched; H, female recently hatched (with a matrix of empty embryo sacs attached to the pleopods); M, male without spermatophore; NS  =  not significant; SM, male with spermatophore; TM, total males; TF, total females.

* =  *P* value <0.05; **  =  *P* value <0.01; ***  =  *P* value <0.001.

Two samples (J2-617-5 and J2-617-8) were collected from a large, high-density aggregation of shrimp at the Von Damm Vent Field. Although they were placed in two separate chambers of the multi-chamber suction sampler, these samples were collected within minutes of each other at the same location and depth (within a 1 m^2^ area) and could not be discriminated spatially. Consequently these two samples were pooled (J2-617-5/8). Unfortunately constraints of expedition logistics precluded a replicate sample from this vent field. Specimens were fixed in 10% buffered seawater formalin for 48 h and stored in 70% isopropanol.

No specific permits were required for the described field studies. No specific permissions were required for these locations/activities. The location is not privately-owned or protected in any way and the field studies did not involve endangered or protected species.

Carapace length (CL) of each shrimp was measured to the nearest 0.1 mm with Vernier callipers from the rear of the eye socket to the rear of the carapace in the mid-dorsal line. This is the standard measure of length for a shrimp and it is used herein as an indication of body size because it avoids errors associated with measuring a flexible abdomen [25].

The sex of each shrimp was determined under a Leica MZ8 dissecting microscope (*sensu* 8). For all males, the presence/absence of a spermatophoric mass was recorded. All females were categorised as either: brooding (brooding embryos on pleopods 1–4); hatched (with a matrix of empty embryo sacs attached to the pleopods); or female (neither brooding nor hatched).

The ovaries were dissected from the female shrimp and where oocyte size allowed, individual oocytes were removed from each ovary under a Leica EZ4 HD dissecting microscope. Images of oocytes were captured using a Leica EZ4 HD dissecting microscope. Packing of oocytes in ovaries often results in irregular oocyte shapes in *Rimicaris hybisae*. Consequently oocytes were laid flat and measured directly, rather than from histological sections, to ensure maximum cross-sectional areas were recorded (*sensu* [15]). Where female specimen numbers and condition allowed, the feret diameters and areas of 100 oocytes were measured in 30 females per sample (9 females for J2-620-32) using ImageJ. Feret diameter was used to standardise variations in oocyte shape. Images of oocytes were calibrated with measurements of a graticule slide at identical magnification.

**Figure 1:**
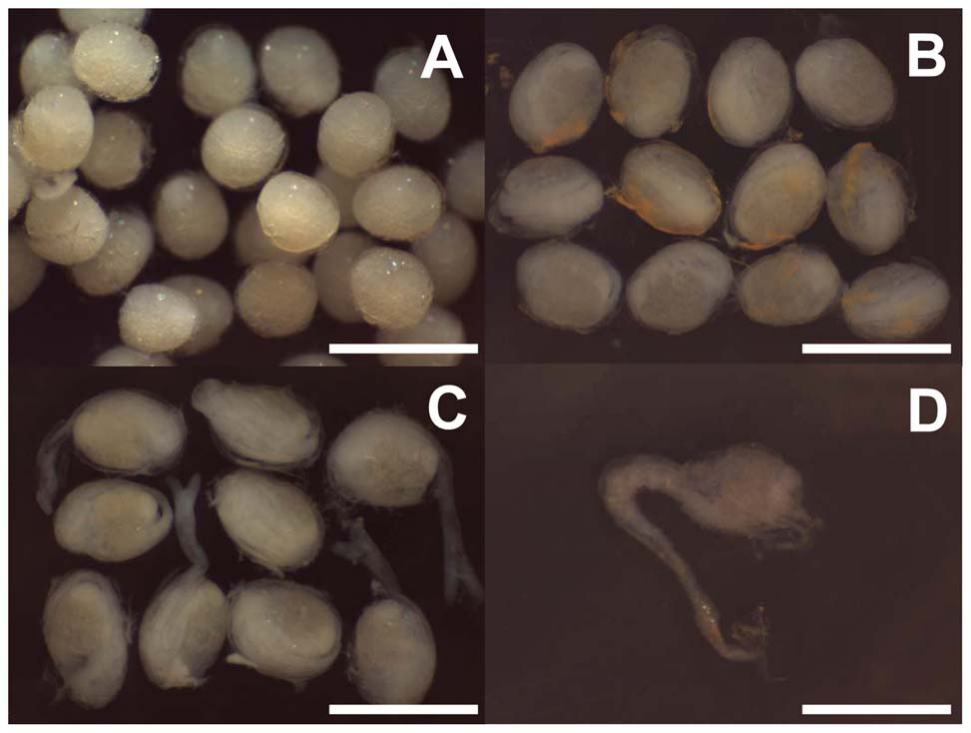
*Rimicaris hybisae*: Embryo stages and larvae. (A) Early-stage embryos; (B) mid-stage embryos; (C) late-stage embryos with larvae ready to hatch; (D) hatched larva. Scale bars = 1 mm.

Broods of embryos were removed from the pleopods of brooding females under a Leica MZ8 dissecting microscope. Within each brood, all embryos had developed synchronously and were at the same stage of development. The developmental stage of each brood was scored on the basis of morphological features (*sensu* [16]): early-stage embryos without features; mid-stage embryos with clear body differentiation; late-stage embryos with clear larval features (e.g. separation of the abdomen from the cephalothorax and developed eyes), including hatched larvae (Figure 1). Numbers of embryos per brood were counted to determine minimum realised fecundity (*sensu* [26]). Although it was not possible to guarantee that embryo batches were complete, embryos were attached firmly to each other and the mothers' pleopods (within which they were enclosed) and broods remained in tact post-sampling. Size-specific fecundity was calculated as number of embryos divided by carapace length (Table 2).

**Table 2:** Average minimum realised fecundity and embryo sizes of caridean shrimp from hydrothermal vents and cold seeps (updated from Ramirez-Llodra & Segonzac, 2006).

Species	*Alvinocaris muricola* (n = 9)	*Alvinocaris stactophila* (n = 55)	*Alvinocaris stactophila* (n = 65)	*Alvinocaris lusca* (n = 1)	*Alvinocaris markensis* (n = 1)	*Chorocaris chacei* (n = 1)	*Mirocaris fortunata* (n = 30)	*Rimicaris exoculata* (n = 2)	*Rimicaris hybisae* (n = 562)	*Rimicaris hybisae* (n = 397)
Site	Congo Basin (seep)	GoM: Brine Pool IMB (seep)	GoM: Brine Pool MMB (seep)	Galapagos Rift (vent)	MAR: Lucky Strike (vent)	MAR: Lucky Strike (vent)	MAR: Lucky Strike (vent)	MAR: Snake Pit & TAG (vent)	MCSC: Beebe (vent)	MCSC: Von Damm (vent)
Depth (m)	3113–3150	500		2500	1690	1690	1690	3480–3650	4944–4972	2294
CL (mm) Mean ± SD	20.7±2.3	3.77	3.91	11.45	13	16.8	7.16±0.18	17.05	10.2±1.5	13.9±2.3
Minimum realised fecundity (embryos) Mean ± SD	3130±1180.9	147	98	407	2007	2510	174.7±22.8	912	341.7±146.2	1054.2±229.8
Size-specific fecundity Mean ± SD	149.1±48.0	39	25	35	154	149	24.3	53	30.6±11.8	68.0±13.2
Mean embryo size (mm)	0.66×0.55	0.80	0.79×0.57	0.50×0.34	0.66×0.52	-	0.70×0.49	0.72×0.62	0.64×0.48	0.63×0.48
Reference	Ramirez-Llodra & Segonzac 2006	Copley & Young 2006	Copley & Young 2006	Van Dover et al. 1985	M. Segonzac, unpublished data	Ramirez-Llodra et al. 2000	Ramirez-Llodra et al. 2000	Williams & Rona 1986; Ramirez-Llodra et al. 2000	This paper	This paper

CL, carapace length; GoM, Gulf of Mexico; IMB, inner mussel bed site; MMB, middle mussel bed site; MAR, Mid-Atlantic Ridge; MCSC, Mid-Cayman Spreading Centre; N, number of females analysed; Size-specific fecundity, embryos/mm CL; SD, standard deviation.

To determine mean embryo size, a sub-sample of ten broods at each developmental stage from both vent fields was selected at random. Embryos were laid flat and images of the embryos were captured using a Leica EZ4 HD dissecting microscope. The greater and lesser diameters of 100 embryos per brood were measured using ImageJ.

Frequencies of males and females in samples were tested for significant variation from a 1∶1 sex ratio using χ^2^ test with Yates' correction for one degree of freedom. In analyses of population structure and size-frequency distribution of females, brooding and hatched females were pooled as ovigerous females. To correct for variation in the sex ratio between samples, spatial variation in the population structure was assessed by comparing the ratio of ovigerous (brooding and hatched) females to non-ovigerous females, the ratio of brooding to hatched ovigerous females, and the ratio of males with spermatophores vs without (rather than the overall proportions). Frequencies of ovigerous females, brooding females and males with spermatophores were tested for significant variation from a 1∶1 ratio between vent fields (Table 3) and between pairwise combinations of samples within the Beebe Vent Field (Table 4) using χ^2^ test with Yates' correction for one degree of freedom. Population structure was examined using the size-frequency distribution of 959 individuals (Table 5).

**Table 3:** *Rimicaris hybisae*: Spatial variation in population structure between the Beebe and Von Damm vent fields, January 2012.

	Beebe	Von Damm	Ratio Beebe: Von Damm	χ^2^ (1 df)	Significance
Proportion of females ovigerous in samples	54.2%	42.6%	1.27∶1	13.47	***
Proportion of ovigerous females brooding	83.9%	70.6%	1.19∶1	13.22	***
Proportion of males with spermatophore	45.2%	30.0%	1.51∶1	18.23	***

Ovigerous was defined as brooding or hatched; proportion of females ovigerous refers to the ratio of ovigerous females to all females in samples.

*** =  *P* value<0.001

**Table 4:** *Rimicaris hybisae*: Spatial variation in population structure within the Beebe Vent Field, January 2012.

Sample	J2-613-19	J2-613-24	J2-620-32	J2-619-15
J2-613-19	-	914.90^***^	**0.03 NS**	71.08^***^
J2-613-24	4.30*	**-**	43.67^***^	57.70^***^
J2-620-32	4.75*	19.19***	-	5.11^*^
J2-619-15	11.02***	19.19***	5.07*	-

Results of χ^2^ (1 df) analyses on proportions of females ovigerous (brooding or hatched; bold text) and proportions of males with spermatophores (italic text) in samples.

NS  =  not significant; *  =  *P* value<0.05; **  =  *P* value<0.01; ***  =  *P* value<0.001

**Table 5:** *Rimicaris hybisae*: Variation in body size (carapace length), January 2012.

	Carapace length (mm)					
	Males			Females		
Vent Field	All	Without spermatophore	With spermatophore	All	Non-ovigerous	Ovigerous
Beebe	10.1 (9.0–10.7)	9.7 (8.7–10.6)	10.2 (9.5–10.9)	10.6 (9.8–11.3)	9.9 (8.3–10.8)	10.9 (10.4–11.6)
Von Damm	14.9 (13.2–15.0)	13.8 (12.6–14.5)	14.8 (14.2–15.1)	14.7 (12.9–15.6)	13.3 (11.5–14.9)	15.3 (14.8–15.8)

Carapace length is shown as median (inter-quartile range).

## Results

### Population structure in January 2012

The gonads of *Rimicaris hybisae* are paired organs laying over the digestive gland of the cephalothorax. Of the 959 specimens of *Rimicaris hybisae* examined, 393 were identified as male (41%), resulting in an overall sex ratio that deviated significantly from 1∶1 (393 males; 566 females) (Table 1). All males examined had only testes and all females studied have only ovaries. Of the 393 males, 152 (39%) were carrying spermatophores. Of the 566 females, nearly half (277, 49%) were either brooding embryos (218, 39%) or had hatched larvae recently (59, 10%).

The specimens ranged in body size (carapace length) from 5.2 mm (male, Von Damm) to 19.4 mm (male, Von Damm). The carapace length of the smallest male identified (5.2 mm, Von Damm) was less than that of the smallest females (6.2 mm, Beebe Woods); this indicated that any bias in sex ratio was not the result of immature males being misidentified as females. The carapace lengths of the smallest brooding female and smallest male with spermatophores were 8.5 and 6.9 mm respectively (both Beebe Woods).

**Figure 2:**
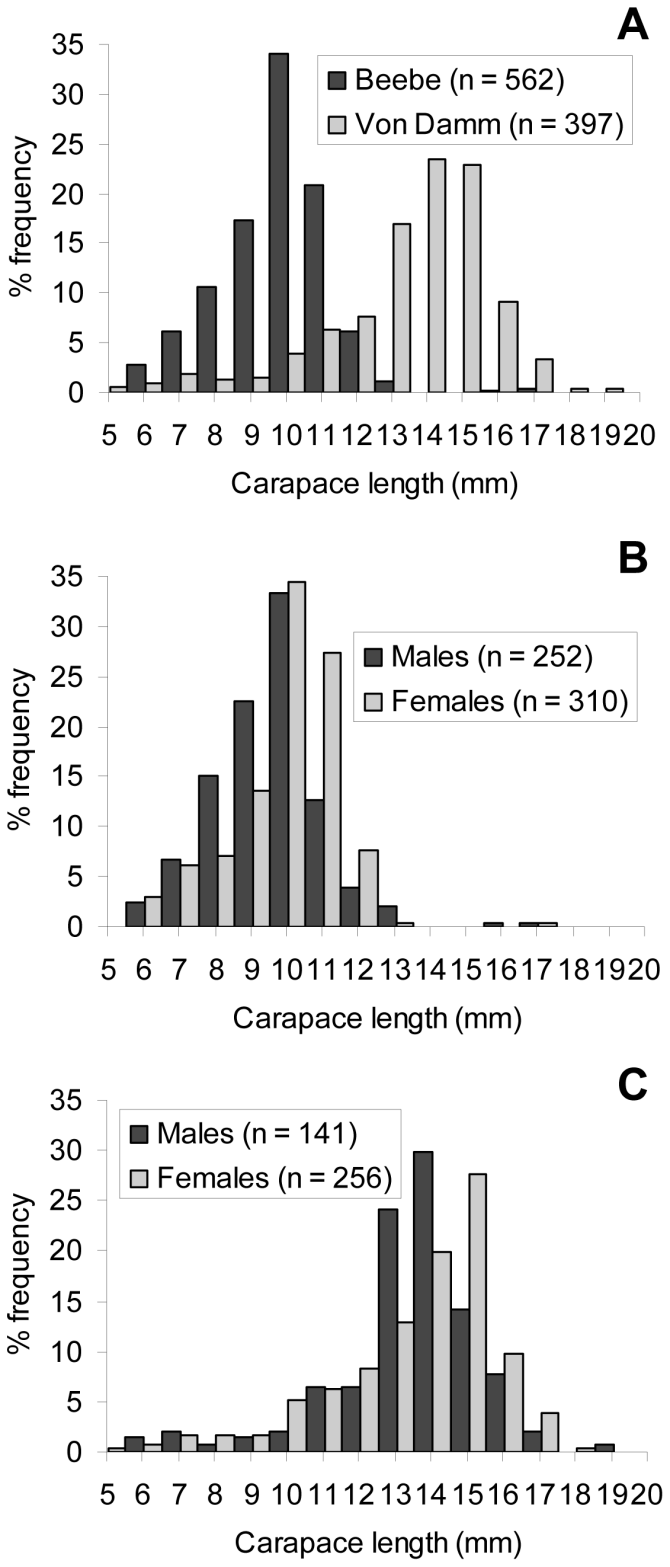
*Rimicaris hybisae*: Size-frequency distribution, January 2012. (A) All specimens; (B) Beebe Vent Field; (C) Von Damm Vent Field. n: no. of individuals measured.

**Figure 3:**
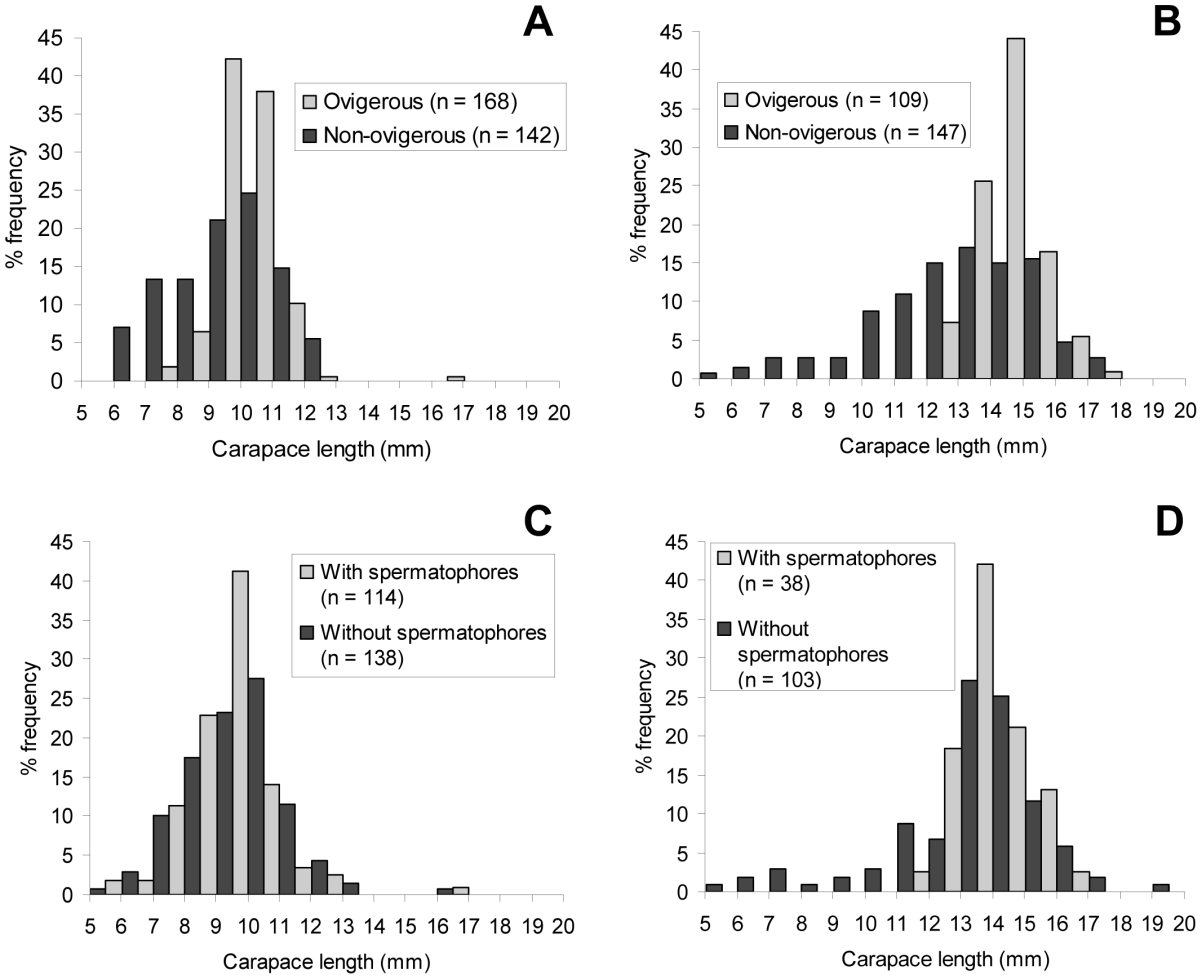
*Rimicaris hybisae*: Size-frequency distribution of females and males, January 2012. (A) Females, Beebe Vent Field; (B) females, Von Damm Vent Field; (C) males, Beebe Vent Field; (D) females, Von Damm Vent Field. n: no. of individuals measured.

The size-frequency distribution of the specimens displayed two modal peaks and a short tail of large specimen sizes (Figure 2A). The carapace lengths of the largest females identified were 17.2 and 18.1 mm at the Beebe and Von Damm Vent Fields respectively. The carapace lengths of the largest males identified were 17.4 and 19.4 mm at the Beebe and Von Damm Vent Fields respectively. The size-frequency distributions of all males and females in January 2012 were significantly different (Mann-Whitney *U*-test, *T* = 168261.5, *p*<0.001). Males were represented throughout the size-frequency distribution of the samples, but there were proportionally fewer large males resulting in a lower median body size in males at both vent fields (Table 5; Figure 2B, C). However, the size-frequency distributions of non-ovigerous (neither brooding nor hatched) females were not significantly different from males (Mann-Whitney *U*-test, *T* = 99797.5, *p*>0.05) and the ratio of males to non-ovigerous females did not deviate significantly from 1∶1 (393 males: 289 non-ovigerous females, χ^2^ = 0, 1 df, p>0.05). Ovigerous (brooding and hatched) females were confined to the peak and tail of large sizes and were significantly larger than non-ovigerous females at both vent fields (Table 5; Figure 3A, B; Mann-Whitney *U*-test, Beebe: *T* = 15731.0, *p*<0.001; Von Damm: *T* = 19167.0, *p*<0.001). Males with spermatophores were significantly larger than males without spermatophores at both vent fields (Table 5; Figure 3C, D; Mann-Whitney *U*-test, Beebe: *T* = 168261.5, *p*<0.001; Von Damm: *T* = 3541.0, *p*<0.001).

The brooded embryos of *Rimicaris hybisae* had a mean size of 0.64×0.48 mm in January 2012 (Table 2). There was no significant difference in embryo sizes between samples from the Beebe and Von Damm vent fields (Table 2; Mann-Whitney *U*-test, *T* = 2250750.0, *p*>0.05). Embryos in the early developmental stage were 0.58±0.04 mm mean greater diameter and 0.46±0.04 mm mean lesser diameter. The mean embryo size in the medium developmental stage was 0.64±0.07 mm greater diameter and 0.48±0.04 mm lesser diameter. Embryos in the most advanced stage were 0.69±0.07 mm mean greater diameter and 0.51±0.05 mm lesser diameter.

### Spatial variation in reproductive features

#### Sex ratio

In January 2012 at the Von Damm Vent Field, 36% of specimens were male, resulting in a sex ratio that deviated significantly from 1∶1 (Table 1). Males represented 45% of individuals sampled from the Beebe Vent Field, resulting in an overall sex ratio that deviated significantly from 1∶1 (Table 1). However, there was significant variation in the sex ratio exhibited in different samples from the Beebe Vent Field (Table 1). Specimens from samples J2-613-19 (Beebe Woods) and J2-619-15 (Shrimp Gulley) did not deviate significantly from a 1∶1 sex ratio. Sample J2-613-24 (Beebe Woods) showed significant female bias (67% of specimens were females), but the ratio of males to non-ovigerous females did not differ significantly from 1∶1. A significant male bias was present in sample J2-620-32 (81% of specimens were males) (Table 1).

#### Population structure

To correct for variation in the sex ratio between samples, spatial variation in the occurrence of ovigerous females was assessed by comparing the ratio of ovigerous (brooding and hatched) females to non-ovigerous females (rather than the overall proportions). A significantly greater proportion of females were ovigerous at the Beebe Vent Field (54%) than at the Von Damm Vent Field (43%; Table 3). The majority of ovigerous females sampled from both vent fields were brooding, as opposed to females that showed evidence of having just hatched their brood (Table 3). However, brooding females represented a significantly greater proportion of ovigerous females at the Beebe Vent Field (84%) compared to the Von Damm Vent Field (71%; Table 3). The majority of males sampled from both vent fields were without spermatophores (Table 3). However, males with a spermatophoric mass accounted for a significantly greater proportion of the sampled male population at the Beebe Vent Field (45%) than at the Von Damm Vent Field (30%; Table 3). As a result of too few data points, it was not possible to determine the correlation between frequencies of ovigerous females, brooding females and males with spermatophores.

**Figure 4:**
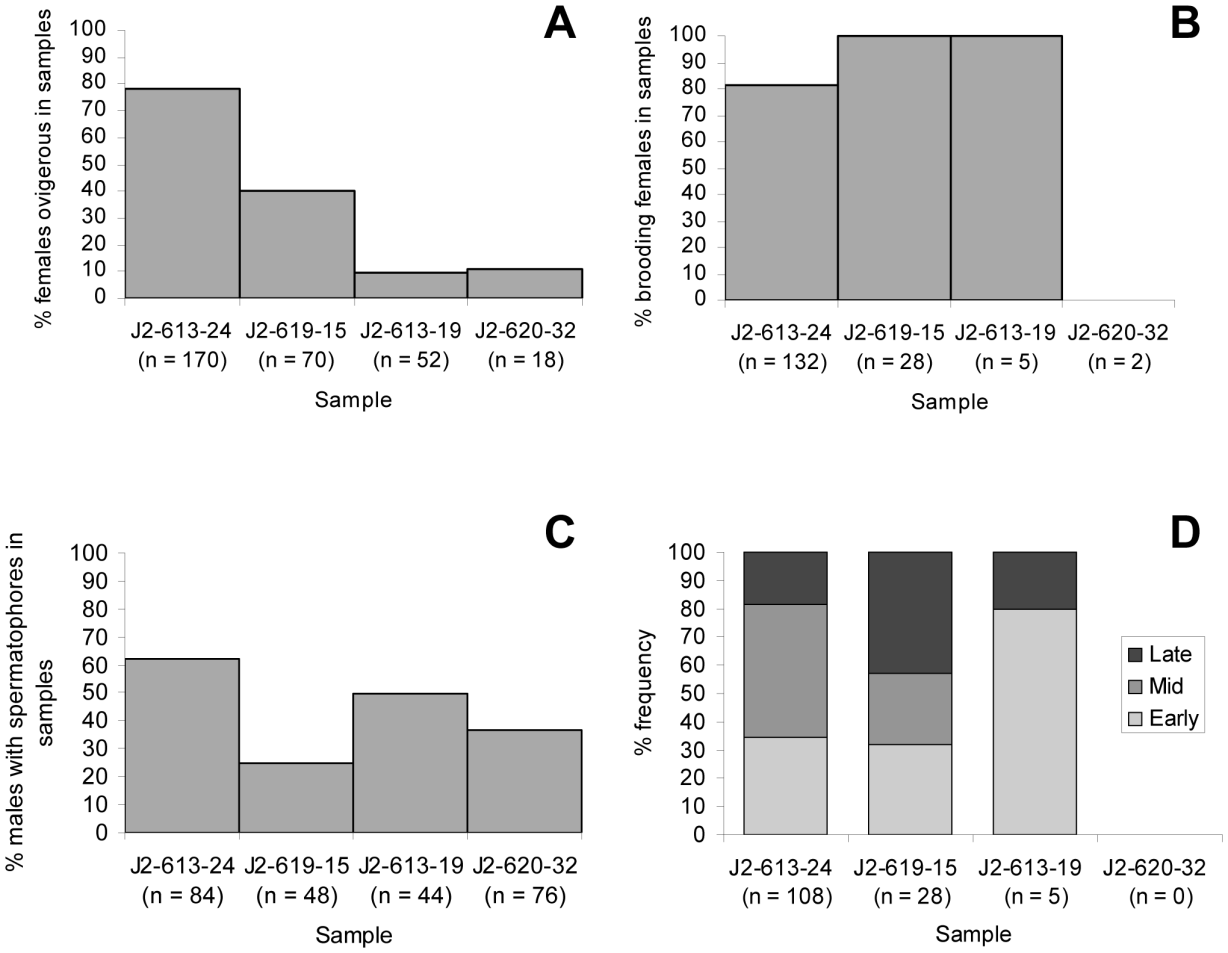
*Rimicaris hybisae*: Spatial variation in samples from the Beebe Vent Field. (A) in proportion of females ovigerous (defined as brooding embryos or hatched) in samples of females; (B) proportion of ovigerous females brooding in samples of ovigerous females; (C) proportion of males with spermatophores in samples of males; (D) proportion of embryos in samples of brooding females at each developmental stage. n: sample size (100%) in each case.

Within the Beebe Vent Field, the highest proportion of ovigerous females was 78% in sample J2-613-24 (Figure 4A). All samples were significantly different from each other in the proportion of ovigerous females with the exception of samples J2-613-19 and J2-620-32 (Table 4). All ovigerous females were brooding in samples J2-613-19 and J2-619-15, whereas 81% were brooding in J2-613-24 and all had hatched in J2-620-32 (Figure 4B). Brooding females accounted for a significantly greater proportion of ovigerous females at J2-619-15 than J2-613-24 (Figure 4B; χ^2^ = 5.31, 1 df, *p*<0.05), and at J2-613-24 than J2-620-32 (Figure 4B; χ^2^ = 4.14, 1 df, *p*<0.05). All other pairwise combinations of samples could not be tested for significant variation from a 1∶1 ratio as a result of expected frequencies of zero.

The highest proportion of males with a spermatophoric mass was 62% in sample J2-613-24 (Figure 4C). All samples from the Beebe Vent Field were significantly different from each other in the proportion of males with spermatophores (Table 4, Figure 4C). As a result of too few data points, it was not possible to determine the correlation between frequencies of ovigerous females, brooding females and males with spermatophores.

### Size-frequency distribution of shrimp

Overall, shrimp sampled from the Von Damm Vent Field were significantly larger than those from the Beebe Vent Field (Table 5; Figure 2; Mann-Whitney *U*-test, *T* = 281949.0, *p*<0.001). Although the January 2012 samples as a whole displayed two modal peaks and a short tail of large sizes, the peak of larger sizes was absent among samples from the Beebe Vent Field, where smaller shrimp were sampled with proportionally greater frequency (Figure 2). The peak of larger sizes was prominent among the samples from the Von Damm Vent Field, where smaller shrimp were sampled with proportionally lower frequency (Figure 2).

Both males and females were significantly larger at the Von Damm Vent Field than the Beebe Vent Field (Table 5; Figure 2; Mann-Whitney *U*-test, males: *T* = 42656.0, *p*<0.001; females: *T* = 104412.5, *p*<0.001). Females were represented throughout most of the size-frequency distribution at both vent fields, but there were proportionally greater large females at the Von Damm Vent Field (Figure 2; Mann-Whitney *U*-test, Beebe: *T* = 61750.5, *p*<0.001; Von Damm: *T* = 25602.5, *p*<0.025). Non-ovigerous females were significantly smaller at the Beebe Vent Field than the Von Damm Vent Field (Table 5; Figure 3; Mann-Whitney *U*-test, *T* = 12744.5, *p*<0.001), as were ovigerous females (Table 5; Figure 3; Mann-Whitney *U*-test, *T* = 24202.0, *p*<0.001). The smallest carapace length exhibited by an ovigerous female at the Von Damm Vent Field was 13.3 mm, compared with 8.5 mm at the Beebe Vent Field). Males with and without spermatophores were both significantly larger at the Von Damm Vent Field than the Beebe Vent Field (Table 5; Figure 3; Mann-Whitney *U*-test, males with spermatophores: *T* = 5026.5, *p*<0.001; males without spermatophores: *T* = 18127.0, *p*<0.001). The smallest carapace length shown by a male with a spermatophore was 12. 9 mm at the Von Damm Vent Field (compared with 6.9 mm at the Beebe Vent Field).

The size-frequency distributions of non-ovigerous (neither brooding nor hatched) females were not significantly different to males at the Beebe Vent Field (Table 5; Figure 2B, 3A; Mann-Whitney *U*-test, *T* = 26586.0, *p*>0.05) and the ratio of males to non-ovigerous females did not deviate significantly from 1∶1 (138 males: 142 non-ovigerous females, χ^2^ = 0, 1 df, *p*>0.05). There was a significantly lower proportion of large non-ovigerous females compared with males at the Von Damm Vent Field (Table 5; Figure 2C, 3B; Mann-Whitney *U*-test, *T* = 22364.5, *p*<0.001). However, the ratio of males to non-ovigerous females did not deviate significantly from 1∶1 (141 males: 147 non-ovigerous females; χ^2^ = 0, 1 df, *p*>0.05).

**Figure 5:**
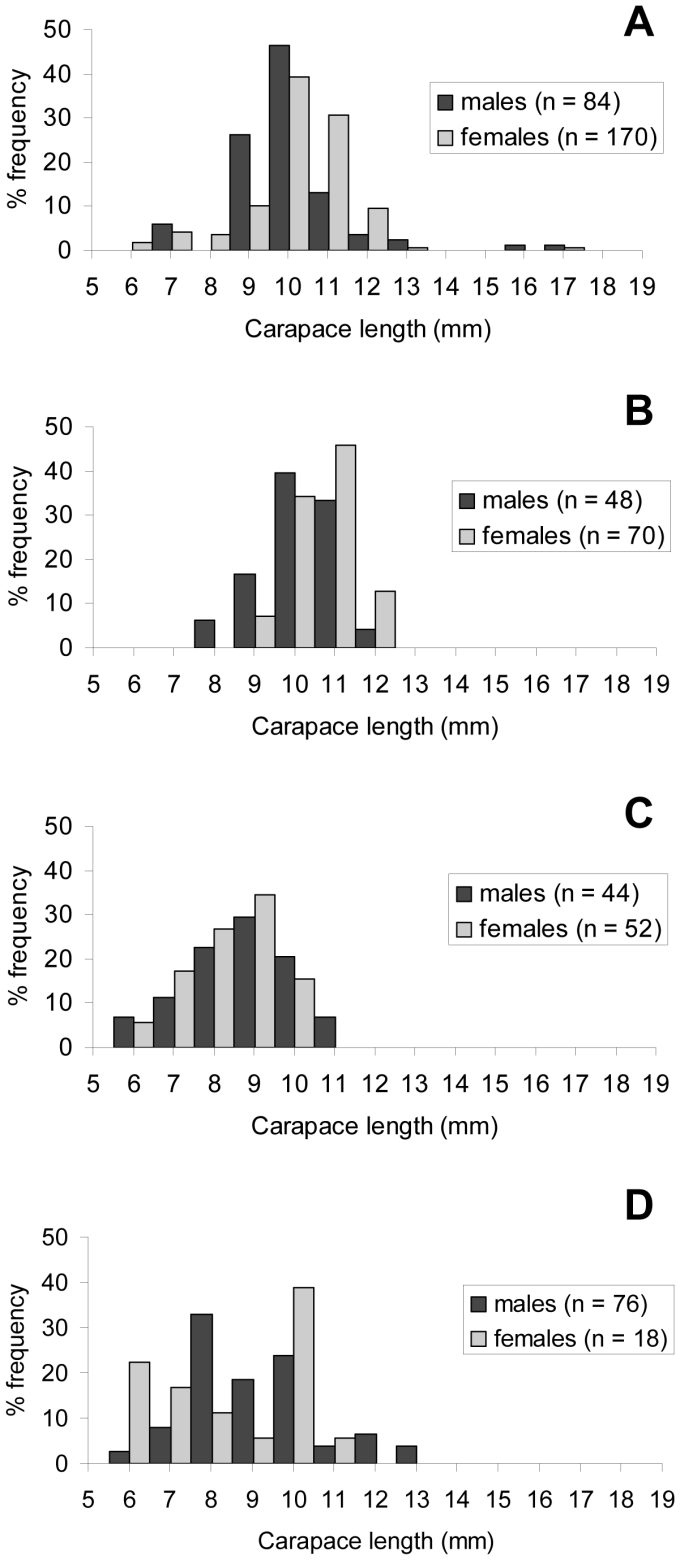
*Rimicaris hybisae*: Size-frequency distribution within the Beebe Vent Field, January 2012. (A) Sample J2-613-24; (B) sample J2-619-15; (C) sample J2-613-19; (D) sample J2-620-32. n: no. of individuals measured.

Overall, there was significant variation in the size-frequency distributions of shrimp collected from different locations within the Beebe Vent Field (Figure 5; Kruskal-Wallace multisample test, *H* = 150.857, 3 df, *p*<0.001). There was also significant variation in the size-frequency distributions of each sex between samples from the Beebe Vent Field (Kruskal-Wallace multisample test, males: *H* = 42.8, 3 df, *p*<0.001; females *H* = 107.0, 3 df, *p*<0.001).

Shrimp in sample J2-613-19 displayed the smallest median size for each sex (males: CL 9.1 mm, IQR 8.2–10.2; females: CL 9.5 mm, IQR 8.3–9.9). However, the median sizes of males and females in sample J2-613-19 were not significantly different from those in sample J2-620-32 (Figure 5; Dunn's Multiple Comparison Test, *p*>0.05). The median sizes and size-frequency distributions of males and females were not significantly different within sample J2-613-19 (Figure 5; Mann-Whitney *U*-test, *T* = 2388.0, *p*>0.05; Kolmogorov-Smirnof two-sample test, *D* = 0.05, *p*>0.05).

The median sizes and size-frequency distributions of males and females were not significantly different within sample J2-620-32 (Figure 5; Mann-Whitney *U*-test, *T* = 723.0, *p*>0.05; Kolmogorov-Smirnof two-sample test, *D* = 0.12, *p*>0.05). However, the number of females was low. The median sizes were CL 9.2 (IQR 8.4–10.5 mm) for males and 9.3 mm (IQR 7.4–10.3) for females.

Sample J2-613-24 exhibited a peak of large females and significantly greater median sizes for females than males (Figure 5; males: CL 10.3 mm, IQR 9.7–10.8; females: CL 10.8 mm, IQR 10.2–11.4; Mann-Whitney *U*-test, *T* = 8779.5, *p*<0.001). The distribution of sizes between males and females was significantly different (Kolmogorov-Smirnof two-sample test, *D* = 0.25, *p*<0.01).

Shrimp in sample J2-619-15 exhibited the largest median size for both males (CL 10.6 mm, IQR 10.1–11.2) and females (CL 11.2 mm, IQR 10.1–11.2) but the median size of males in J2-613-24 (CL 10.3 mm, IQR 9.7–10.8) was not significantly different (Figure 5; Dunn's Multiple Comparison Test, *p*<0.05). Sample J2-613-15 exhibited a peak of large females and significantly greater median sizes for females than males (Figure 5; Mann-Whitney *U*-test, *T* = 2267.5, *p*<0.01). The distribution of sizes between males and females was significantly different (Kolmogorov-Smirnof two-sample test, *D* = 0.30, *p*<0.05).

### Fecundity

The embryos in broods of *Rimicaris hybisae* formed a dense mass attached to pleopods 1–4 underneath the female abdomen. The broods were orange in colour and visible in video footage from the Beebe and Von Damm vent fields in January 2012. The greatest minimum realised fecundity determined among 218 brooding females examined from January 2012 was 1707 in an individual from the Von Damm Vent Field with a carapace length of 16.6 mm.

Specimens from the Von Damm Vent Field exhibited significantly greater fecundity than those from the Beebe Vent Field (Table 2; Von Damm vs J2-613-19, vs J2-613-24, vs J2-619-15, Kruskal-Wallace multisample test, *H* = 139.8, 3 df, *p*<0.001; Dunn's Multiple Comparison Test, *p*<0.005). The median number of embryos brooded by females from the Von Damm Vent Field was 1062 (IQR 931–1191). The median number of embryos brooded by females from the Beebe Vent Field ranged from 122 (IQR 20–304, J2-613-19, Beebe Woods) to 385 (IQR 357–439, J2-619-15, Shrimp Gulley). Differences in the fecundities of females among the samples from the Beebe Vent Field were not significant (Dunn's Multiple Comparison Test, *p*>0.05).

**Figure 6:**
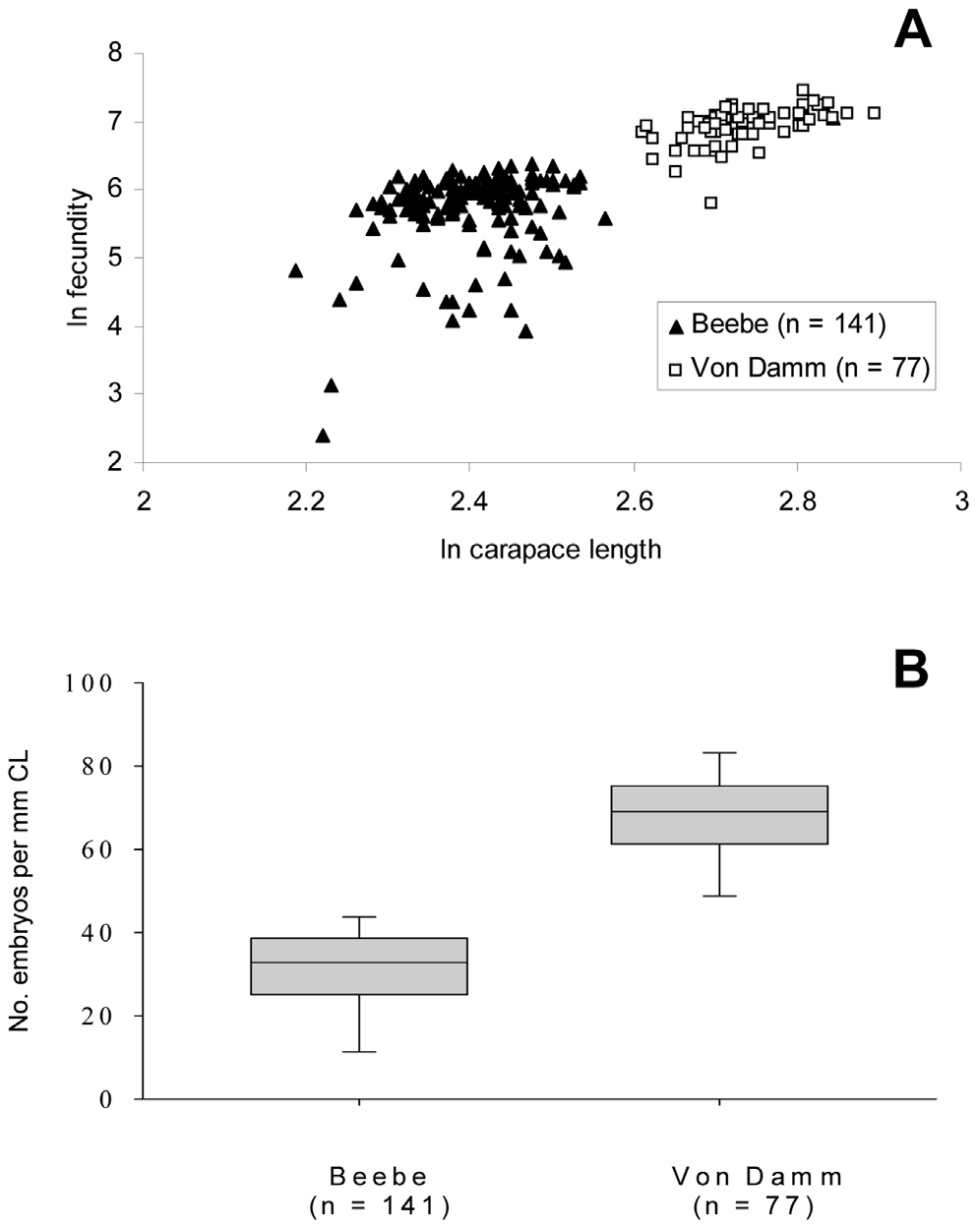
*Rimicaris hybisae*: Fecundity (no. of embryos on pleopods), January 2012. (A) Variation of log*_e_*-transformed minimum realised fecundity with log*_e_*-transformed carapace length; (B) corrected for body size (carapace length). n: no. of individuals measured.

Minimum realised fecundity correlated positively with carapace length (Figure 6A; Spearman correlation, *r* = 0.77, *p*<0.0001). The slope of the relationship between log*_e_*-transformed fecundity and log*_e_*-transformed body size (carapace length) did not differ significantly among specimens from the Von Damm Vent Field and the Beebe Vent Field (Figure 6A; *F* = 0.06, *p*<0.05). Overall, size-specific fecundity (related to carapace length) ranged from 1.2 to 102.8 embryos mm^−1^. Although brooding females at the Von Damm Vent Field carried more embryos per mm carapace length than those at the Beebe Vent Field (Figure 6B), this difference could therefore be attributable to the larger body sizes of brooding females at Von Damm and a non-linear relationship between fecundity and body size, which appears to be consistent across the two vent fields.

### Embryo developmental stages

Within each brood of the 218 brooding females analysed, the embryos had developed synchronously and were all at the same stage of development (early, mid or late; Figure 1). However, there was no evidence of synchrony between the broods of different females. Overall, the majority of broods (48%) were in a medium developmental stage. One third (34%) exhibited an early stage of development, whereas advanced-stage broods were the minority (19%). The majority of broods at both the Von Damm and Beebe vent fields were in mid-stage development (60% and 41% respectively), and there were a greater proportion of broods at the early-developmental stage compared to the late stage (30% vs 10% Von Damm; 36% vs 24% Beebe).

In sample J2-613-19, 80% of broods were at the early stage and the remaining 20% were at the late stage (Figure 4D). In contrast, 47% of broods were at the mid-stage in sample J2-613-24; 34% were early-stage and 19% were late-stage (Figure 4D). Late-stage broods accounted for 43% of broods in sample J2-619-15, with 32% and 25% at the early and mid-stage broods respectively (Figure 4D). There were no brooding females in sample J2-620-32.

### Oocyte size-frequency distribution

**Figure 7:**
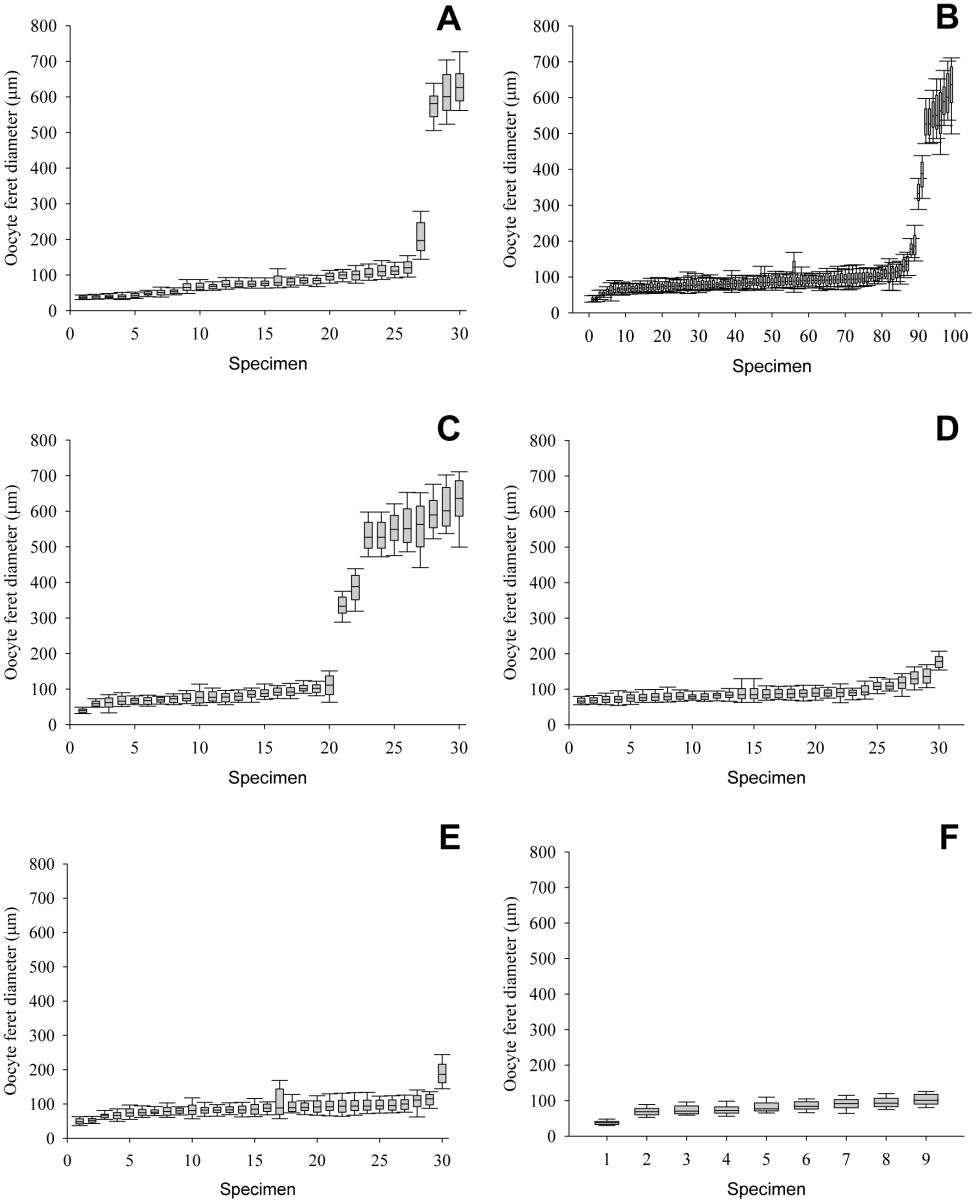
*Rimicaris hybisae*: Spatial variation in oocyte size-frequency distribution at the Beebe (B–F) and Von Damm (A) vent fields, January 2012. (A) Von Damm Vent Field; (B) Beebe Vent Field (all samples); (C) sample J2-613-24; (D) sample J2-619-15; (E) sample J2-613-19 (F) sample J2-620-32.

Females examined from January 2012 contained oocytes with feret diameters ranging from 21 µm (non-ovigerous female, CL 12.3, Von Damm) to 823 µm (non-ovigerous female, CL 16.7, Von Damm) (Figure 7). Specimens examined from both vent fields included both ovigerous (brooding and hatched) and non-ovigerous females.

The median oocyte size in individuals from the Von Damm Vent Field was 82 µm (IQR 59–109). Three distinct oocyte sizes were apparent among females collected from the Von Damm Vent Field in January 2012 (Figure 7A). The larger oocytes belonged exclusively to large, non-ovigerous females (CL 14.4–17.5 mm). These specimens exhibited median oocyte sizes ranging from 581 µm (IQR 545–602) to 626.5 µm (IQR 589–665). The small oocytes belonged to ovigerous and non-ovigerous females (CL 11.6–17.2 mm); these specimens displayed a range of median oocyte sizes from 37 µm (IQR 33–41) to 120.5 µm (IQR 105.5–137). One individual (non-ovigerous female, CL 14.96 mm) showed a median oocyte size outside both these ranges (196.5 µm, IQR 169–246).

The median oocyte size in 99 individuals from the Beebe Vent Field was 86 µm (IQR 71–109). Three distinct oocyte sizes were apparent among females collected from the Beebe Vent Field in January 2012 (Figure 7B). Females examined in samples J2-619-15, J2-613-19 and J2-620-32 contained relatively small oocytes with feret diameters between 26 and 262 µm (Figure 7). Median oocyte sizes in individuals from sample J2-619-15 ranged from 66.5 µm (IQR 61–74.5) to 178 µm (IQR 162–191.5). Median oocyte sizes in the nine females from sample J2-613-19 ranged from 48 µm (IQR 42–57) to 186.5 µm (IQR 162–215.5). The range of median oocyte sizes in females from sample J2-620-32 was 38 µm (IQR 33–41.5) to 101 µm (IQR 90.5–117). Specimens examined from these samples included both ovigerous and non-ovigerous females. Three distinct oocyte sizes were evident among females from sample J2-613-24 (Figure 7C). Here the larger oocyte sizes belonged to non-ovigerous females (CL 9.2–10.3 mm). These individuals exhibited median oocyte sizes ranging from 527 µm (IQR 496–569) to 636 µm (IQR 586.5–683.5). The mid-size oocytes also belonged to non-ovigerous females (CL 9.6–10.7 mm); these specimens displayed median oocyte sizes ranging from 333 µm (IQR 315.5–358.5) to 388 µm (IQR 353–419.5). The smaller oocytes were from ovigerous and non-ovigerous females (CL 9–12.4 mm). These females revealed median oocyte sizes ranging from 40 µm (IQR 34.5–44) to 111 µm (IQR 84.5–136.5).

Overall, there was significant variation in oocyte sizes between samples (Kruskal-Wallis multi-sample test, *H* = 371.8, 4 df, *p*<0.01) and no evidence of synchrony between samples. There were significant differences in oocyte sizes between every pairwise comparison of all five samples (Dunn's Multiple Comparison Test, *p*<0.05).

## Discussion

### General features of reproduction in *Rimicaris hybisae*

The gonads of *Rimicaris hybisae* are similar to those of other caridean shrimp [16], [13], [27]. *Rimicaris hybisae* exhibits sexual dimorphism and is a gonochoric species, consistent with all other alvinocaridid species studied to date.

The maximum size of males analysed was larger than the maximum size of females of *Rimicaris hybisae*. Nevertheless, in January 2012 females exhibited a significantly larger median body size and greater size range than males with proportionally greater large females at both the Beebe and Von Damm vent fields. A larger size in females has been inferred for *Alvinocaris muricola* based on the maximum size of males vs females [16] and a larger size of females is a common feature of caridean shrimp [28]. However, the variance in the size of sexes in this study appears to be the result of spatial variation in the proportion of males and females in samples, rather than sexual dimorphism.

Males with spermatophores were significantly larger in carapace length than males without spermatophores in January 2012. Ovigerous (brooding and hatched) females were significantly larger than non-ovigerous females at both vent fields, whereas the size-frequency distributions of non-ovigerous females were not significantly different from males and the ratio of males to non-ovigerous females did not deviate significantly from 1∶1. The larger size of ovigerous females has been reported previously for *Alvinocaris stactophila* from the Brine Pool cold seep [15]. A greater size of ovigerous females may be advantageous for embryo production where fecundity correlates positively with body size, as in *Rimicaris hybisae*. A positive correlation between body size and fecundity has been determined for every alvinocaridid species in which these variables have been examined (*A. muricola* and *A. stactophila* [13], [15]; *Mirocaris fortunata* [16]; *R. hybisae*, this study). This feature of carideans is a result of space availability between the pleopods for attachment of embryos [29].

The maximum oocyte size measured in *Rimicaris hybisae* females in January 2012 was 823 µm, which is greater than maximum oocyte sizes recorded for other alvinocaridid shrimp (*R. exoculata*: 500 µm [13], 601 µm [17]; *Alvinocaris muricola*: 515 µm [16]). However, the smaller mature oocytes in the range exhibited by *R. hybisae* (Figure 7) fall within the range recorded previously in the literature. Although it is possible that mature oocytes attain a greater size in *R. hybisae* than in other alvinocaridids for which information is available, an alternative explanation is that the true size range of mature oocytes has not been captured yet in other species, given smaller sample sizes from which data have been generated in other species.

Embryos had developed synchronously within (but not between) broods in January 2012. The developmental stages of embryos observed in *Rimicaris hybisae* were similar to those described for *Alvinocaris muricola* [16]. One experimental study found that the embryos of two brooding specimens of *Alvinocaris* sp. hatched after 73 and 79 days of rearing, with complete hatching of the brood over 7 and 14 days [30]. Nevertheless, it remains to be determined how long alvinocaridid females incubate their young for *in situ*, how long embryos take to develop from one stage to the next and whether these features are variable between different environmental conditions. Further experimental studies in the laboratory and *in situ* would be required to elucidate these knowledge gaps.

**Figure 8:**
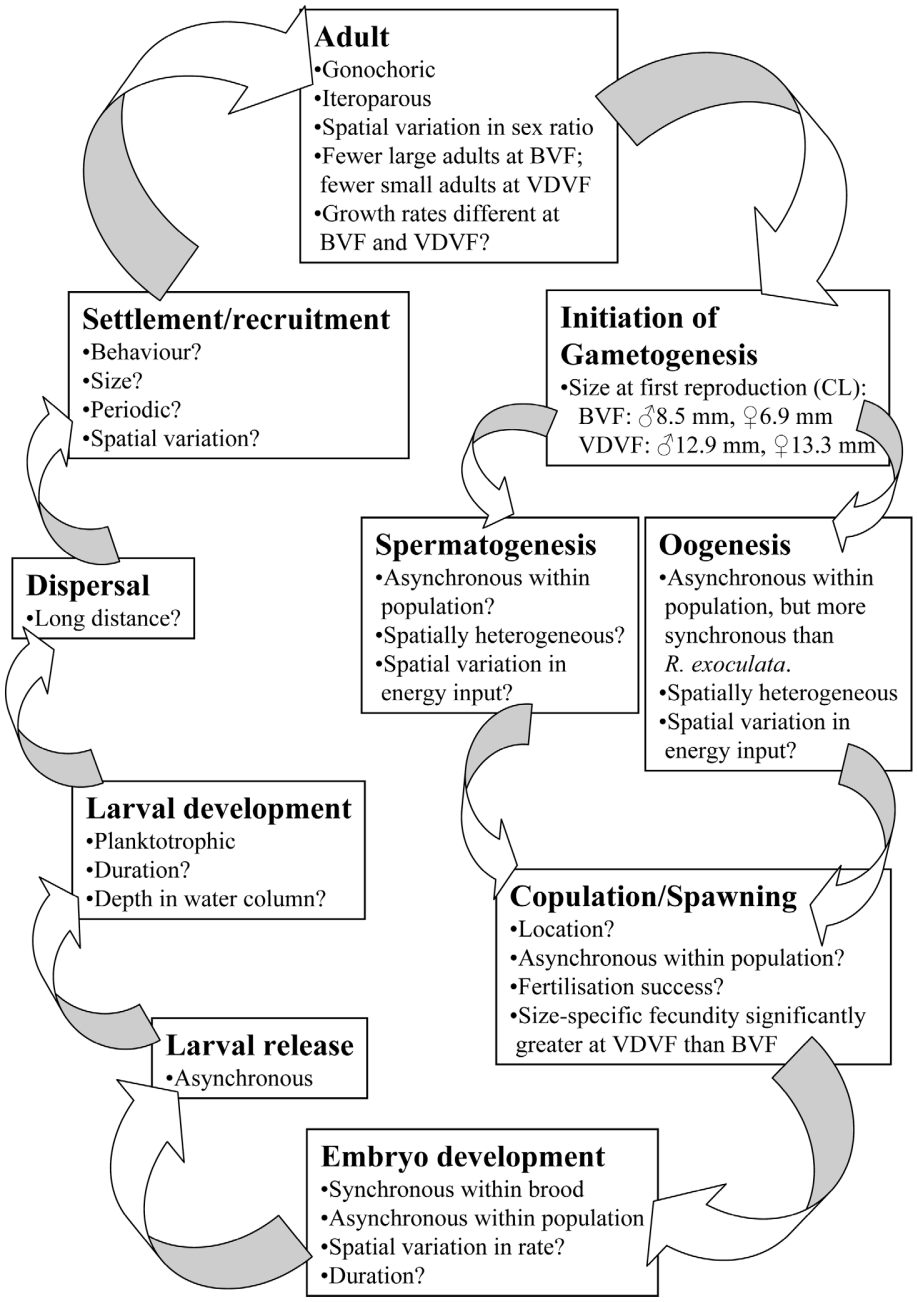
*Rimicaris hybisae*: Inferred life-history (sensu Tyler & Young, 1999). Stages are in bold. BVF, Beebe Vent Field; CL, carapace length; VDVF, Von Damm Vent Field.

Tyler & Young [2] reported that a complete life cycle had yet to be elucidated for a single vent or seep species. Following their template for a generalised marine invertebrate life cycle, what is now known, inferred, or unknown in gametogenesis, copulation/spawning, embryo development, larval ecology, dispersal and recruitment are summarised for *Rimicaris hybisae* in Figure 8.

Embryos of *Rimicaris hybisae* were variable in size but there was no spatial variation in embryo size. Embryos ranged in size from 0.42–0.76 mm (greatest diameter) with a mean size that was consistent with those reported in other alvinocaridid species to date (Table 2). The small size of embryos revealed in all the alvinocaridids for which data are available is indicative of planktotrophic larvae with extended development [27]. This hypothesis is supported by biochemical and experimental studies for other species [30], [31], [32], [33], [34], [35], [36], [37], [38], [39]. A long larval duration could facilitate extended dispersal between patchily distributed chemosynthetic environments and promote genetic diversity and colonisation of new vents [40], [41], [42]. Biochemical studies would be required to confirm the trophic ecology of *R. hybisae* during the larval (and adult) phase.

It has been suggested that upward vertical movement during the larval phase could explain the broad geographic distribution of certain species with planktotrophic larvae entrained in deep-water currents [4]. Vent shrimp postlarvae have been found to be present in midwater above MAR vents and to extend great distances laterally from known chemosynthetic sites [43]. However, the period and depth of planktotrophic development in the water column has not been specified for a single alvinocaridid species to date. The larval phase remains one of the least known stages in the life-cycle of most deep-sea species [6], yet elucidating the larval ecology of a species is a prerequisite to understand patterns of reproduction and recruitment. Studies of larval tolerances, lifespan and mortality during dispersal combined with hydrographic and phylogeographic data are required to address this significant gap in the life-cycle of *Rimicaris hybisae* and other alvinocaridid species.

### Spatial variation in population structure and reproductive features

In January 2012, the sampled population of *Rimicaris hybisae* at the Von Damm Vent Field was dominated by large females and a lesser proportion of slightly smaller males. The sex ratio was significantly female-biased but the ratio of males to non-ovigerous females did not differ significantly from 1∶1. However, there was a significantly smaller proportion of large non-ovigerous females compared with males. Large ovigerous females accounted for a high proportion of the female population. Males with a spermatophore represented quite a high proportion of the male population but were only present in the larger size classes (CL>12 mm).

Overall, the sampled population at the Beebe Vent Field was similar to that at the Von Damm Vent field (see above), but with a significantly greater proportion of ovigerous and brooding females and males with spermatophores. The sex ratio at the Beebe Vent Field was also significantly female-biased overall, although males represented a greater proportion of the population in comparison with the Von Damm Vent Field. There was no significant difference in size between males and non-ovigerous females and the ratio of males to non-ovigerous females did not differ significantly from 1∶1. However, individual samples from the Beebe Vent Field were heterogeneous. Recognition of this spatial heterogeneity is crucial to consider in any attempts to infer temporal patterns from limited spatial samples.

Ovigerous female *Rimicaris hybisae* and males with spermatophores were present with the greatest frequency in sample J2-613-24. This sample was dominated by ovigerous females and the sex ratio was significantly female-biased. However, the ratio of males to non-ovigerous females did not differ significantly from 1∶1. Females were significantly larger than males and both sexes were significantly larger than those in samples J2-613-19 and J2-620-32.

Large male and female *Rimicaris hybisae* were present with the greatest frequency in sample J2-619-15. The sex ratio in this sample did not differ significantly from 1∶1. Ovigerous females were present with the second greatest frequency here and females were significantly bigger than males. Males with spermatophores were present with the lowest frequency in this sample.

Ovigerous female *Rimicaris hybisae* were present with the lowest frequency in samples J2-613-19 and J2-620-32 and the size of males and females were not significantly different from each other in these samples. The sex ratio in sample J2-620-32 was significantly male-biased, whereas it did not differ significantly from 1∶1 in J2-613-19.

The population of *Rimicaris hybisae* showed a significant clear bias towards females in the sample from the Von Damm Vent Field and sample J2-613-24 from Beebe Woods. These samples also contained the greatest proportion of ovigerous females. Both samples were collected from large, high-density aggregations of shrimp. Ramirez-Llodra & Segonzac [16] described a clear bias towards females in *Alvinocaris muricola* in the cold-seep site north of Regab in the Congo Basin. Copley & Young [15] identified a specific distribution of males and females within the Brine Pool mussel bed, with ovigerous females avoiding the sulphidic or anoxic extremes in the environment. Hydrothermal vents exhibit fine-scale heterogeneity in physico-chemical conditions [44]. Environmental variables within shrimp aggregations may influence the distribution of ovigerous females, resulting in a spatially heterogeneous pattern of reproductive development in *R. hybisae*, as found in other vent taxa [45], [46]. Further sampling and collection of environmental data would be required to test this hypothesis.

Shrimp at the Von Damm Vent Field were significantly larger than shrimp at the Beebe Vent Field both overall and within each population category. The smallest ovigerous female (CL 6.9 mm) and male with a spermatophore (CL 8.5 mm) at the Beebe Vent Field were much smaller than the smallest ovigerous female (CL 13.3) and male with spermatophore at the Von Damm Vent Field (CL 12.9 mm). Size at the onset of maturity is considered a key life-history parameter that should also reflect the longevity and life-time investment in reproduction of a species [26]. Assuming these data represent the minimum size of sexual maturity in *Rimicaris hybisae*, males and females at the Beebe Vent Field may reach maturity at smaller sizes than their counterparts at the Von Damm Vent Field.

Size-specific fecundity in *Rimicaris hybisae* falls within the range of values reported in other alvinocaridid species (Table 2). Fecundity was significantly positively correlated with body size in *R. hybisae* and there was no significant difference in the slope of the relationship between log*_e_*-transformed fecundity and log*_e_*-transformed carapace length at the two vent fields. There is no clear relationship between the size-specific investments in reproduction in alvinocarids and phylogeny, depth or environment (vent versus seep) [16]. Some variation in measured fecundity may result from females losing eggs during collection [3] but variation in fecundity may also result from variations in reproductive success, which may be affected by environmental conditions [15]. Environmental factors such as pressure, availability of photosynthetically-derived nutrition, temperature, and/or fluid chemistry may vary between the two vent fields which are considerably different in depth. However, a non-linear relationship between fecundity and carapace length may result from volume effects either for the developing ovary or in the space around the pleopods on which embryos develop. Consequently, the greater size-specific fecundity of females at Von Damm may be attributable to their larger body size rather than environmental conditions, given the homogeneity of regression between log*_e_*-transformed carapace lengths and log*_e_*-transformed fecundities across both vent fields.

The oocyte size-frequency distributions exhibited significant variation between samples and all stages of developing oocytes were present amongst females in January 2012. Most females had gonads containing previtellogenic oocytes (<100 µm) and early vitellogenic oocytes (>100 µm). However, several large non-ovigerous females from the Von Damm Vent Field and sample J2-613-24 contained large vitellogenic oocytes. These data indicate iteroparous reproduction in *Rimicaris hybisae*. However, the variation observed was less than that recorded among individual female *R. exoculata* from TAG [17], suggesting a greater degree of synchrony in the oocyte development of *R. hybisae* than reported for *R. exoculata*.

Embryo development was clearly asynchronous between females of *Rimicaris hybisae*, indicating that larval release may also be asynchronous for the population as a whole. Environmental variability may potentially affect every reproductive process, resulting in embryo development proceeding at different rates within a vent field.

Video from a previous cruise (NOAA ‘Okeanos Explorer’) to the Von Damm Vent Field in August 2011 showed no evidence of brooding *Rimicaris hybisae*, whilst individuals in video collected during January 2012 showed clear evidence of embryo carrying, suggesting possible periodic reproduction for *R. hybisae*. The collection and analyses of additional samples spanning several seasons of the year are a prerequisite to determine the potential periodicity or even seasonality in reproduction and possibly recruitment of *R. hybisae*.

Periodic reproduction has also been suggested for *Rimicaris exoculata* based on the almost complete absence of ovigerous females in collections from summer months (when most samples have been collected) [47]. However, observations of oocyte size-frequency distributions of females collected in different times of the year indicate iteroparous, asynchronous reproduction and lack of seasonal reproduction in *R. exoculata* from MAR vents [13], [17]. An alternative hypothesis that has been proposed is that ovigerous females stay outside the main populations [13]. However, recent observations of ovigerous females of *R. exoculata* within the main aggregations around high temperature zones at Logatchev in March 2007 [48] refute the latter hypothesis.

Periodic production of eggs has also been proposed for *Mirocaris fortunata* [13], whereas continuous egg production with periodic spawning was suggested for *Alvinocaris muricola* [16]. In contrast, a seasonal pattern of reproduction has been revealed in *A. stactophila* at the Brine Pool cold seep [15] and a few other species with planktotrophic larvae from vent environments (e.g., [14], [49], [50]). Surface productivity may therefore be important for the nutrition of planktotrophic larvae of some vent and seep species, particularly at shallower depths.

## Conclusions

Reproductive features examined in *Rimicaris hybisae* at the Beebe and Von Damm vent fields are consistent with those described previously for other alvinocaridid species, consistent with phylogenetic constraint of such features in vent species. Several gaps remain, however, in understanding the life cycle of this and other alvinocaridid species.

Samples collected from the Von Damm and Beebe vent fields in January 2012 revealed spatial variation in the population structure and reproductive features of *Rimicaris hybisae*. These data highlight a high degree of spatial variability in the reproductive features of a mobile species in the vent environment. The sample from the Von Damm Vent Field and sample J2-613-24 from Beebe Woods exhibited the highest frequencies of ovigerous females and significantly female-biased sex ratios. Any bias in sex ratio was not the result of immature males being misidentified as females. Nevertheless, when the generally large ovigerous females were excluded, there was no significant deviation from a 1∶1 sex ratio. Environmental variables within shrimp aggregations may influence the distribution of ovigerous females, resulting in a spatially heterogeneous pattern of reproductive development in *R. hybisae*, as found in other vent taxa. This hypothesis would require testing with the collection and analysis of further samples with environmental data (temperature, fluid chemistry).

Reproduction in *Rimicaris hybisae* is iteroparous. The oocyte development of *R. hybisae* appears to exhibit a greater degree of synchrony than reported previously for *R. exoculata*. However, embryo development and larval release may be asynchronous for the population as a whole. Analysis of video from a previous cruise to the Von Damm Vent Field in a different season revealed no evidence of ovigerous females. These results suggest a lack of synchrony and possible periodicity in the reproductive development of *R. hybisae*. Any subsequent investigation of temporal variation in the reproductive development of *R. hybisae*, however, needs to take into account the spatial variation also revealed by this study.

Specimens of *Rimicaris hybisae* from the Von Damm Vent Field were significantly larger than specimens from the Beebe Vent Field and may reach maturity at larger sizes than their counterparts at the Beebe Vent Field. Given a possible non-linear relationship between fecundity and carapace length, the larger body sizes of brooding females at Von Damm may result in a greater size-specific fecundity compared with females at the Beebe Vent Field.
